# RNA biology of disease-associated microsatellite repeat expansions

**DOI:** 10.1186/s40478-017-0468-y

**Published:** 2017-08-29

**Authors:** Kushal J. Rohilla, Keith T. Gagnon

**Affiliations:** 1 Department of Biochemistry and Molecular Biology, Southern Illinois University, School of Medicine, Carbondale, Illinois USA; 2 Department of Chemistry and Biochemistry, Southern Illinois University, Carbondale, Illinois USA

**Keywords:** Repeat expansion disease, Microsatellite, Tandem repeats, RNA, Splicing, Transcription, Transport, Export, Turnover, Translation, Mechanism, Therapeutics, Myotonic dystrophy, DM1, DM2, Huntington's disease, HD, C9ORF72, C9FTD/ALS, Amyotrophic lateral sclerosis, Spinocerebellar ataxia, SCA, Fragile X, SBMA, FXTAS

## Abstract

Microsatellites, or simple tandem repeat sequences, occur naturally in the human genome and have important roles in genome evolution and function. However, the expansion of microsatellites is associated with over two dozen neurological diseases. A common denominator among the majority of these disorders is the expression of expanded tandem repeat-containing RNA, referred to as xtrRNA in this review, which can mediate molecular disease pathology in multiple ways. This review focuses on the potential impact that simple tandem repeat expansions can have on the biology and metabolism of RNA that contain them and underscores important gaps in understanding. Merging the molecular biology of repeat expansion disorders with the current understanding of RNA biology, including splicing, transcription, transport, turnover and translation, will help clarify mechanisms of disease and improve therapeutic development.

## Introduction

In 1991 it was discovered that a microsatellite sequence expansion is the cause of two distinct neurological disorders, Fragile X syndrome (FXS) [303] and spinal bulbar muscular atrophy (SBMA), or Kennedy's disease [167]. Since then, simple repeat sequence expansions have been associated with over twenty more neurological disorders [166, 300, 333] (Table 1). What has been learned is that microsatellite expansions may cause disease in multiple ways. For nearly all of these neurological disorders, however, disease includes production of RNA that contains the aberrant repeat expansion sequence. Accordingly, the leading disease mechanisms involve repeat expansion RNA-mediated sequestration of critical RNA-binding proteins and translation of repeat expansion RNA into toxic repetitive polypeptides.

**Table 1:** Microsatellite repeat expansion disorders

Disorder	Repeating unit	Genomic location	Gene name	Normal length	Pathogenic length	Expanded repeats result in:				Repeat discovery & references
						Gene silencing	xtrRNA transcription	xtrRNA proteins	xtrRNA foci	
FXS/FRAXA^†^	CGG	5’UTR	*FMR1*	6-55	200+	Yes*	No	No	No	[114, 161, 230, 303]
SBMA^†^	CAG	Coding	*AR*	9-36	38-62	No	Yes	Yes*	L .D.	[167]
DM1	CTG	3’UTR	*DMPK*	5-37	50-10000	No	Yes*	Yes	Yes*	[193]
HD	CAG	Coding	*HTT*	10-35	35+	No	Yes	Yes*	L.D.	[192]
SCA1	CAG	Coding	*ATXN1*	6-35	49-88	No	Yes	Yes*	L.D.	[232]
FRAXE	CCG	5'UTR	*AFF2*	4-39	200-900	Yes*	No	No	No	[152]
DRPLA	CAG	Coding	*ATN1*	6-35	49-88	No	Yes	Yes*	L.D.	[155]
SCA3	CAG	Coding	*ATXN3*	12-40	55-86	No	Yes	Yes*	Yes	[140]
SCA2	CAG	Coding	*ATXN2*	14-32	33-77	No	Yes	Yes*	L.D.	[127, 253]
FRDA	GAA	Intron	*FXN*	8-33	90+	Yes*	Yes / No	No	No	[30]
SCA6	CAG	Coding	*CACNA1A*	4-18	21-30	No	Yes	Yes*	L.D.	[337]
EPM1	CCCCGC CCCGCG	Promoter	*CSTB*	2-3	30-80	Yes*	Yes / No	No	No	[170]
SCA7	CAG	Coding	*ATXN7*	7-17	38-120	No	Yes	Yes*	L.D.	[57]
OPMD	GCG	Coding	*PABPN1*	6-10	12-17	No	Yes	Yes*	No	[23]
SCA8	CTG	3’UTR	*ATXN8*	16-34	74+	No	Yes	Yes*	Yes*	[156]
SCA12	CAG	5’UTR	*PPP2R2B*	7-28	66-78	No	Yes*	No	No	[121]
SCA10	ATTCT	Intron	*ATXN10*	10-20	500-4500	No	Yes	?	Yes*	[205]
SCA17	CAG	Coding	*TBP*	25-42	47-63	No	Yes	Yes*	L.D.	[224]
DM2	CCTG	Intron	*CNBP*	10-26	75-11000	No	Yes	?	Yes*	[184]
FXTAS/FXPOI	CGG	5’UTR	*FMR1*	6-55	55-200	No	Yes	Yes*	Yes*	[143, 287]
HDL2	CTG/CAG	3'UTR/antisense	*JPH3*	<50	50+	No	Yes	?	Yes*	[120]
SCA31	TGGAA	Intron	*TK2/BEAN*	0	45+	No	Yes	Yes*	Yes*	[256]
SCA36	GGCCTG	Intron	*Nop56*	3-14	650+	No	Yes	?	Yes*	[153]
C9FTD/ALS	GGGGCC	Intron	*C9ORF72*	2-25	25+	No	Yes	Yes*	Yes*	[59, 248]
FRA7A	CGG	Intron	*ZFN713*	5-22	85+	No	Yes* / No	No	No	[209]
FRA2A	CGG	Intron	*AFF3*	8-17	300+	No	Yes* / No	No	No	[210]

Disorders are listed in order of the year they were discovered, with the appropriate references relating to their discovery. This table highlights known RNA biology for each disease with respect to xtrRNA transcription, translation, and formation of nuclear focal aggregates

Dagger symbol (†) indicates that athough the CAG repeat for SBMA was discovered first, the CGG repeat for FXS was published first. Asterisk (*) indicates the most likely repeat-associated disease mechanism(s) for that disorder. *L.D.* length-dependent, *SBMA* Spinal-Bulbar Muscular Atrophy, *EPM1* Progressive Myoclonus Epilepsy 1 (Unverricht–Lundborg Disease), *FXS/FRAXA* Fragile X Syndrome, *DM* Myotonic Dystrophy, *HD* Huntington’s Disease, *SCA* Spinocerebellar Ataxia, *FRAXE* Fragile X E Syndrome, *DRPLA* Dentatorubral-Pallidoluysian Atrophy, *FRDA* Friedreich Ataxia, *OPMD* Oculopharyngeal Muscular Dystrophy, *FXTAS* Fragile X–Associated Tremor/Ataxia Syndrome, *FXPOI* Fragile X-Associated Primary Ovarian Insufficiency, *HDL2* Huntington’s Disease-Like 2, *C9FTD/ALS C9ORF72*-Associated Frontotemporal Dementia and Amyotrophic Lateral Sclerosis, *FRA7A* CGG Expansion at Fragile Site 7A, *FRA2A* CGG Expansion at Fragile Site 2A

Tremendous progress has been made in understanding the metabolism of expanded tandem repeat-containing RNA (xtrRNA). Nonetheless, various gaps in our understanding of the underlying molecular biology and pathology remain, which in turn limits identification of promising therapeutic approaches. The goal of this review is to help address these gaps by discussing the potential impact of xtrRNA on cellular RNA metabolism. We begin with an overview that covers microsatellite origin, evolution, and expansion. We then follow xtrRNA through its life cycle, beginning with transcription and continuing through splicing, folding, protein interactions, localization, turnover, and translation. We rationalize the logic of current molecular disease models, note where important mechanistic information is lacking, and emphasize new pathways to consider for mechanistic insight. We use this discussion to also highlight areas where therapeutic intervention may be useful.

## Origin and expansion of microsatellites in human disease

### Simple tandem repeat sequences in the human genome

Microsatellite sequences comprise approximately 3% of the human genome, about twice as much as protein coding sequence [1, 171]. Microsatellites, interchangeably known as simple or short tandem repeats (STRs), are usually defined as simple sequence motifs of one to six nucleotides that are contiguously repeated at least a few times [24, 69]. Microsatellites occur throughout the genome, but are predominantly found in noncoding promoters, introns, 5' and 3' untranslated regions (UTRs), and intergenic regions [236, 257, 291]. Intergenic microsatellites seem to fit neutral evolution models, although not perfectly [69], and are among the most variable genomic sequences [32]. Therefore, they serve as the basis for forensic DNA analyses and as markers for population genetics studies [24, 61, 65, 236].

The origin and evolution of microsatellites is incompletely understood. They may have derived from simple and repetitive sequence motifs found in mobile genetic elements, such as non-LTR (long terminal repeat) retrotransposons like *Alu* and L1 [6, 51, 95]. Transposable elements have colonized the human genome extensively and their remains have undergone mutation and replication, providing the starting material for simple tandem repeats [69]. The dense repetitive sequence of centromeres and telomeres is proposed to originate from incorporation of mobile genetic elements during early eukaryotic evolution [87, 304]. Small sequence duplications of these simple sequences can further produce microsatellites with multiple repeats. The STRs that are expanded in Friedreich ataxia (FRDA) and myotonic dystrophy type 2 (DM2), for example, have been traced to an *Alu* element origin [41, 163]. In contrast, *de novo* genesis by events like random mutation, replication slippage, and duplication of unique sequence may also account for the birth of microsatellite sequences [28, 69]. STRs have been shown to have positive roles in evolution, such as bacterial resistance to antibiotics and circadian clock adaptation to the environment in *Neurospora crassa* and *Drosophila melanogaster* [83, 133, 211, 258, 279, 307]. The placement of microsatellite sequences in and near regulatory and coding regions of the genome also implicate them in control of gene expression and genetic interactions [133, 331].

### Expansion of simple tandem repeats

There are several distinct mechanisms that can contribute to the expansion of naturally occurring microsatellites. In this section we provide a brief overview of these mechanisms. Many excellent reviews on this topic are cited in this section and recommended for further reading (see [84, 130, 149, 206, 212, 260, 297, 331, 333]).

A major source of microsatellite expansion in dividing cells is DNA replication, although mitotic recombination is also recognized as a contributing factor [84, 149, 212, 242]. During replication, repetitive sequences can cause problems at the replication fork and result in fork reversals or template switching, which can insert extra repeats [76, 144, 149, 212]. At the strand level, polymerase slipping can cause expansions in the leading or lagging strand [84, 137, 144, 149]. Repeats may also induce imperfect Okazaki fragment ligation and add repeats in the lagging strand [81, 93, 278]. The pathway followed for expansion of a repeat has been proposed to be a balancing act between several factors [84, 149, 212]. These include relative repeat length, the stability and types of non-canonical structures the repeat sequence can form, and nearby flanking sequences. After repeat sequences are added to one or both strands, the daughter strands reanneal. Misalignment and slippage will occur and extra sequences will bulge out to form non-canonical (non-B-form) structures like hairpins or quadruplexes [237, 331]. If these structures persist to the next round of replication, or if they undergo flawed repair, they can result in permanent expansions [130, 149, 212, 260, 297]. During DNA recombination, which repairs single-end or double-strand breaks, unequal crossing over or template switching can cause misalignments and introduction of additional repeats [208, 242, 306].

Repeat expansion events are intimately tied to the repair of non-canonical DNA structures and DNA damage. Multiple DNA damage control pathways have been implicated, including mechanisms that replace DNA bases, like base excision repair (BER) or nucleotide excision repair (NER), especially as sources for repeat expansion in non-dividing cells [206]. However, mismatch repair (MMR) has been argued to be a primary driver of repeat expansion [75, 106, 130, 260, 271]. MMR expands repeats through recognition and processing of unusual DNA structures, such as small bulges and hairpins [260], via the enzyme MutSβ (MSH2-MSH3 complex) [130, 260, 334]. The processing and damage rectification steps are carried out by MutSβ and associated proteins, including the MutLα (MLH1-PMS2 complex) or MutLγ (MLH1-MLH3 complex) endonucleases that help remove DNA lesions [106, 130, 241]. Polymerases like Polβ are then recruited, which can insert extra repeats due to flawed priming or templating [33, 190].

An important question is how repeats are able to expand out of control, sometimes into the hundreds or thousands of perfect tandem copies, without accumulating significant interruptions? Microsatellites that are evolutionarily neutral, typically in intergenic regions, become highly mutable when they exceed thresholds above just a few tandem repeats [68, 95, 320]. Therefore, the likelihood of remaining as a perfect tandem repeat without interruption is expected to decrease with tandem repeat length. This suggests that accumulation of large expansions must either occur quickly, before mutations can accumulate, or their disruption must be guarded against [320]. Genic regions of the genome, where all currently known disease-associated repeat expansions occur [31, 236] (Table 1), seem to enjoy special favor through positive evolutionary selection processes that protect sequence fidelity [191, 236, 284]. However, it seems unlikely that this would contribute significantly to large repeat expansions. For example, non-repetitive codons would presumably be preferred and selected over unstable repeat codons.

Mechanisms have been proposed that could provide large expansions in a single step, including template switching replication models where repeats are already sufficiently large enough [225, 266] and out-of-register synthesis during homologous recombination-based repair of double-strand breaks (DSBs) [212, 242, 249, 250, 283]. One intriguing mechanism for rapid and large repeat accumulation is break-induced replication (BIR) [148, 176]. BIR is a homologous recombination pathway that can rescue collapsed or broken replication forks [195]. It is induced when a replisome collides with a broken single-end DSB [189]. BIR is also believed to be selective for structure-prone or GC-rich repeats that are long enough to form stable structures [148]. In this mechanism of expansion, stable structures would cause fork reversals. Resolution of these four-way junction structures would result in a one-ended DSB. To restart the fork, the one-ended DSB invades the sister chromatid to form a D-loop, but likely does so out-of-register because of the repetitive sequence, thus leading to expansion. While this BIR study was performed in yeast, the results are expected to translate to human cells [176].

Incremental expansions, such as those caused by MMR, are typically on the order of 1-3 repeats at a time [260]. Could these events generate large uninterrupted expansions? Rapid accumulation of expansions via MMR or other DNA damage repair pathways might be facilitated by transcription across the repeat. It has been shown that transcription is required for expansion of the CGG repeat in a mouse model of FXS [2, 333]. Several studies have shown that transcription at repeat expansions is associated with repeat instability, possibly via formation of DNA-RNA hybrids, or R-loops [180, 181, 183, 195, 223, 246, 333]. It is possible that these events could allow DNA damage. One report has shown a correlation between R-loop formation and replication fork stalling, offering a familiar mechanism for repeat expansion through DNA replication [86, 109]. Alternative mechanisms might involve oxidation of free DNA strands, or simple misalignment upon strand reannealing, to signal DNA damage repair [180]. The latter model suggests that GC-rich or structure-prone repeats would be more susceptible to expansion during transcription, which might explain why transcription levels alone are not predictive of expansion [333]. Thus, cycles of transcription and R-loop formation might accelerate repeat expansion for structure-prone repeats via ongoing DNA damage repair [180]. A cell-based model where transcription levels or R-loop formation could be controlled, repeat sequence and size altered, expansions monitored, and DNA damage repair mechanisms systematically tested (perhaps building on HeLa cell models recently described [187]) might allow more direct testing of these ideas.

A common theme among sources of repeat instability and expansion is DNA metabolism associated with strand separation and reannealing at microsatellite sequences. These events can lead to formation of non-canonical structures and recruitment of DNA damage responses that ultimately and inadvertently add more repeats. Thus, mechanisms meant to maintain and protect the genome can also lead to large tandem repeat expansions and cause human disease [11, 130, 333].

### Microsatellite repeat expansion disorders

Since it was first discovered that microsatellite expansions can cause disease, at least two dozen microsatellite repeat expansion disorders have been subsequently reported (Table 1). The latest discoveries are autism spectrum disorders caused by expansions in fragile 7A (FRA7A) and fragile 2A (FRA2A) fragile site loci [209, 210]. Comparing and contrasting these disorders can highlight several trends. Almost half of the microsatellite expansion disorders result from CAG trinucleotide expansions, mostly occurring in coding exons. All STRs for known repeat expansion disorders are GC-rich except for the trinucleotide GAA repeat of FRDA and the ATTCT and TGGAA pentanucleotide repeats of spinocerebellar ataxia 10 (SCA10) and 31 (SCA31)﻿, respectively. In this review we focus on large microsatellite repeat expansions that are transcribed into RNA, a feature that is shared by nearly all repeat expansion disorders (Table 1).

**Fig. 1:**
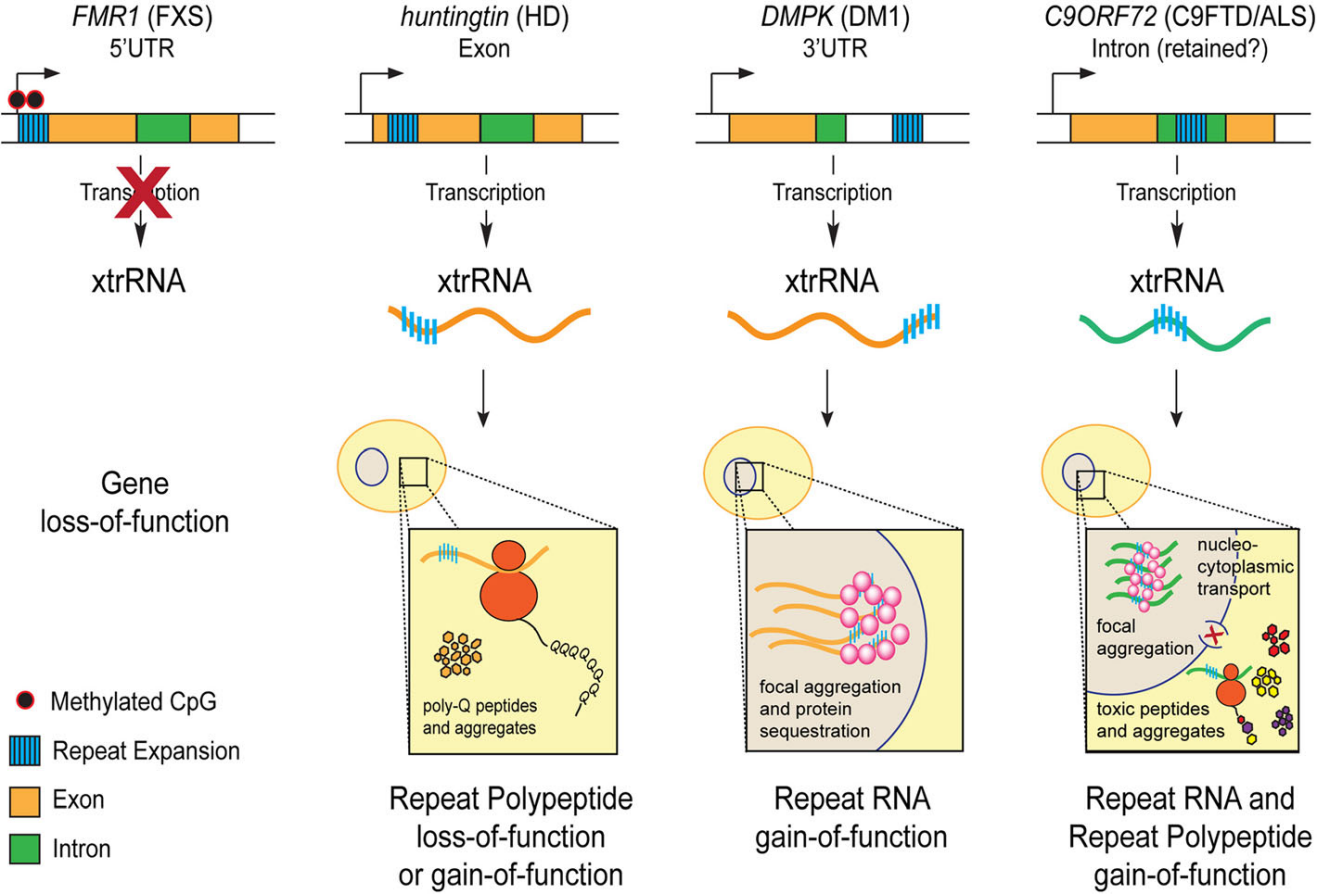
Distinct loss-of-function and gain-of-function mechanisms of disease for various repeat expansion disorders. Repeat expansions can occur in 5’ or 3’ UTRs, exons, or introns. Expanded tandem repeat-containing RNA (xtrRNA) may not be transcribed due to epigenetic silencing, thereby causing loss of gene function. If transcribed, xtrRNA may become trapped in the cell nucleus where it can form focal aggregates and functionally deplete important RNA binding proteins. The xtrRNA may also be exported to the cytoplasm where it can undergo translation to produce repeat-containing polypeptides that disrupt cellular processes. In some cases, xtrRNA can form focal nuclear aggregates and also be translated into repeat-containing polypeptides. Repeat-containing polypeptides can be toxic in multiple ways, including insoluble aggregation, blocking normal host protein function, inhibiting nucleocytoplasmic transport, and disrupting other critical cellular functions

Microsatellite expansions cause disease through two broad molecular mechanisms (Fig. 1): loss-of-function for the associated gene or gain-of-function for the repeat expansion sequence. In loss of function mechanisms, gene expression can be silenced at the transcriptional level, such as by epigenetic modification, resulting in the complete loss of that gene's normal functions [70, 112]. Alternatively, the affected gene may lose function at the protein level by the introduction of unusually long polypeptide tracts in the translated protein product (Fig. 1) [168, 268]. In gain-of-function mechanisms the repetitive polypeptide can take on new roles, such as protein aggregation. Many of these mutant misfolded proteins cannot be degraded efficiently and will accumulate in cellular aggregates or inclusions [48, 168, 332]. Aggregation also tends to sequester proteins and critical cellular components and is taxing on cellular proteostasis [48]. The xtrRNA can also acquire gain of function mechanisms, primarily through interaction with nucleic acid-binding proteins (Fig. 1). The repetitive xtrRNA forms length-dependent focal aggregates in cell nuclei in several diseases [35, 59, 196, 262, 311]. Loss-of-function and gain-of-function mechanisms can result in complicated molecular disease pathologies and some disorders can simultaneously exhibit multiple mechanisms (Table 1).

## Transcription and splicing at simple tandem repeat expansions

### Transcribing repeat expansion sequences

Repeat expansion sequences are known to inhibit or impede RNA Polymerase II (Pol II) initiation or elongation either directly or via induction of a repressed chromatin state [100]. Expansions like the GAA repeat in FRDA [19, 94, 97, 162, 231], the CTG repeat in myotonic dystrophy type 1 (DM1) [25], the GGGGCC repeat in *C9ORF72*-associated frontotemporal dementia and amyotrophic lateral sclerosis (C9FTD/ALS) [108], and the CGG repeat in FXS (also known as FRAXA) [44, 285] have all been implicated in reduced or silenced transcription. For FXS [230, 298], Fragile XE (FRAXE) [18], FRDA [97], FRA2A [210] and FRA7A [209], transcription appears to be blocked or significantly reduced by DNA methylation of the repeat expansion or nearby CpG islands. However, although transcription may be well below basal levels, it is possible that xtrRNA can still contribute to disease in some cases [44].

**Fig. 2:**
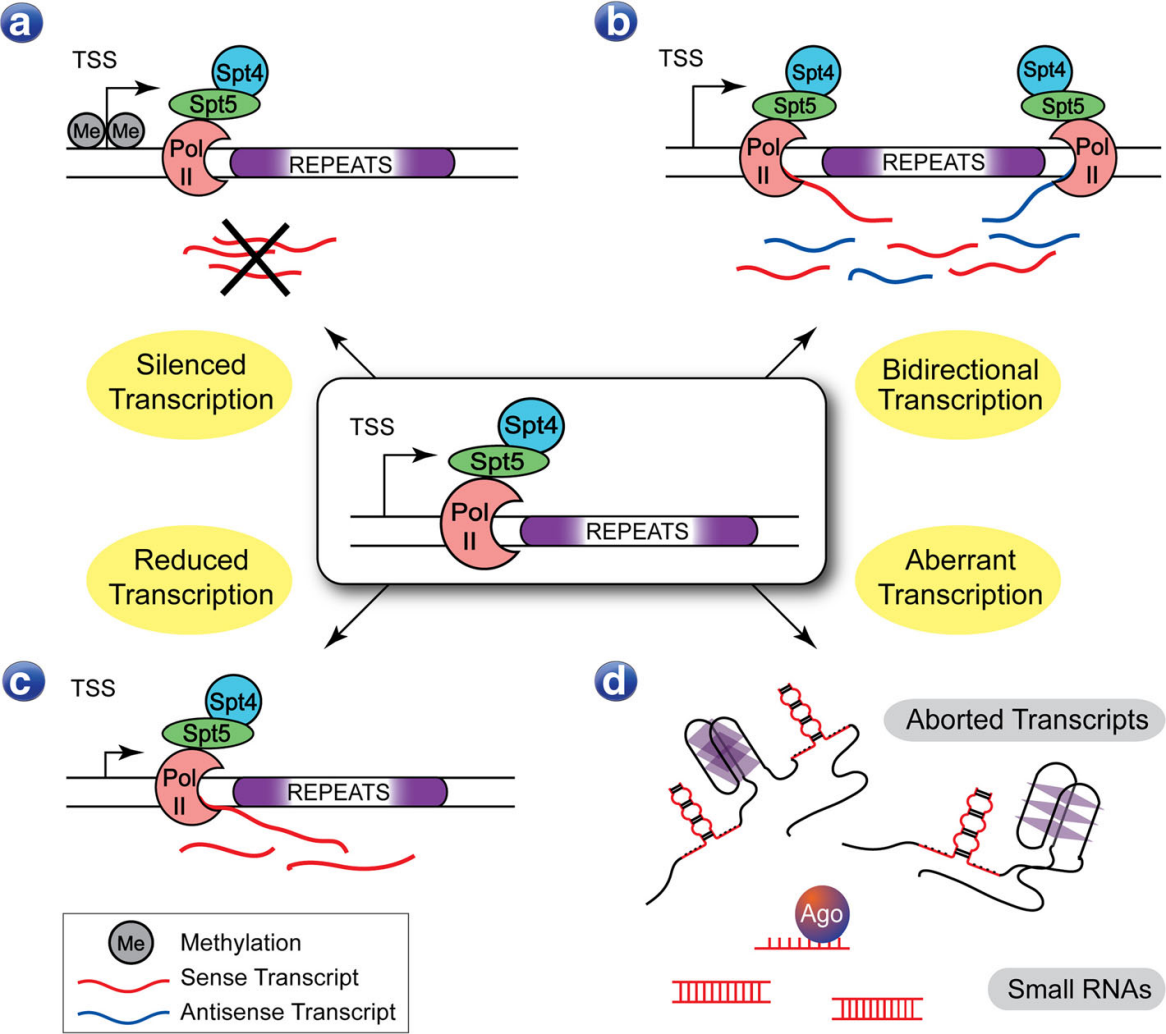
Effects of repeat expansion sequence on transcription. Repeat expansion sequences can perturb transcription by **a** epigenetic silencing, **b** inducing or facilitating bidirectional transcription, **c** reduced transcription kinetics, or **d** generating transcripts that can potentially be processed into small RNAs that could guide degradation or silencing of various complementary RNAs, including the xtrRNA itself

Slowed or stalled transcription across repeat expansions may lead to R-loops, which further slow transcription [123] and inadvertently contribute to deposition of repressive chromatin marks and silence transcription (Fig. 2) [44, 99, 316, 317]. R-loops play important roles in biology, such as immunoglobulin class switching [323], keeping CpG islands unmethylated [91, 254], and defining transcription termination signals [254, 270]. R-loop formation is common in transcription of C-rich template sequences [324], which most disease-associated repeat expansion genomic loci possess. The impact of R-loop formation on disease at repeat expansions is still unclear. Whether R-loop formation will trigger DNA methylation, transcriptional silencing, or other events may be dependent upon a number of factors specific to the affected gene or locus.

### Bidirectional transcription of repeat expansions

Bidirectional transcription has been reported to occur in DM1, C9FTD/ALS, Huntington's disease (HD), spinocerebellar ataxia 8 (SCA8), and Huntington's disease-like 2 (HDL2), among other diseases [26, 40, 126, 312]. Slowed transcription across a repeat may also be able to induce antisense transcription of the non-template DNA strand via R-loop formation [270]. For example, FRDA-associated GAA repeat expansion sequences were shown to initiate transcription and act as promoters in yeast [330]. However, many genes exhibit bidirectional transcription [293] and in microsatellite diseases bidirectional transcription typically initiates outside of the repeat (Fig. 2) [26, 113]. Bidirectional transcription across repeats can also result in double R-loops that amplify repeat instability and accelerate methylation and transcriptional silencing [181, 183, 223]. Antisense transcription can often interfere with transcription of the coding gene [145]. Most relevant to this review is the production of two xtrRNAs from bidirectional transcription and the potential to synthesize repetitive polypeptides from both xtrRNA. For example, in C9FTD/ALS both xtrRNAs form nuclear foci [59, 90, 248, 340] that sequester RNA-binding proteins [47, 175, 217] and are translated into repetitive polypeptides [9, 216], highlighting the importance of bidirectional transcription to molecular disease pathology.

### The role of Supt4h in xtrRNA transcription

Transcribing microsatellite expansions into xtrRNA requires processivity across repetitive sequence tracts that can have very high GC content. The 5,6-dichloro-1-*β*-D-ribofuranosylbenzimidazole (DRB) sensitivity-inducing factor (DSIF), composed of Supt4h and Supt5h proteins (Spt4 and Spt5 in yeast), aids RNA Polymerase II (Pol II) in transcription elongation and transcription rate [305, 308]. The DSIF complex is important for traversing sequences that elicit pausing of RNA Pol II [305] and has been identified as a factor involved in the transcription of RNA containing large simple repeat sequences. For example, transcription of repeat-containing RNA from the *huntingtin* and *C9ORF72* genes significantly decreases when Supt4h is deleted or knocked-down [159, 186]. Supt5h is a conserved transcription factor with a homolog known as NusG in bacteria that is important for elongation and processivity [185, 202]. Supt5h binds directly to the clamp coiled-coil domain of RNA Pol II while Supt4h interacts through contact with Supt5h [17, 119, 201]. Together, the DSIF complex interacts with the DNA template outside of the transcription bubble [17, 50, 151, 185]. Supt4h has a zinc-finger domain that may be important for modulating DNA interactions of DSIF [308], and thereby improve processivity by maintaining RNA Pol II template interaction during periods of extended pausing [50, 151, 309]. Long repetitive sequences prone to formation of secondary structure in the transcription bubble, such as repeat-induced hairpin or R-loop structures, may represent prime sites for pausing or backtracking [251, 260, 333].

DSIF is also used by RNA Pol I to presumably ensure robust transcription of abundant and repetitive ribosomal RNA [122, 309]. It is worth noting that repeat expansions might occur in ribosomal RNA genes but they have either not been characterized or have not been associated with disease [122]. In contrast, RNA Pol III, which only transcribes relatively small noncoding RNA genes, does not interact with the DSIF complex [309]. Thus, transcription is unlikely to be successful if large microsatellite expansions occur in the small RNA genes transcribed by RNA Pol III. These observations may lend some rationale as to why all disease-associated repeat expansions to date are associated with Pol II-transcribed genic regions [7, 31, 236].

### Splicing of xtrRNA

Splicing involves several regulated steps, many accessory factors and the spliceosome, a complex multi-component enzyme. There is currently a lack of mechanistic insight regarding how the splicing apparatus reacts when encountering pre-mRNA containing large repetitive sequence tracts [14]. Since introns can be excessively large while still allowing productive and accurate splicing [263], the size of the repeat expansion itself is not expected to significantly impede splicing. However, transcription rates across microsatellite expansions can be reduced, which can influence alternative splicing [58, 270], and stem loop structures in large pre-mRNA introns have been predicted to affect splicing [263].

Examples of microsatellite repeat expansions modulating splicing include the GAA repeat expansion associated with FRDA. When placed near reporter gene exons or in the first intron of a *frataxin* minigene system, the GAA repeat caused complex splicing defects and accumulation of aberrant splice products [15]. The mechanism proposed involved binding of various splicing factors to the GAA repeat-containing transcripts [15]. In C9FTD/ALS, the intronic GGGGCC repeat has been implicated in splicing by favoring retention of the intron-containing repeat, suggesting a mechanism by which *C9ORF72* xtrRNA can escape to the cytoplasm for translation [227]. Expanded CAG repeats of HD are also linked to production of short alternatively spliced forms of the *huntingtin* mRNA that contain the CAG repeat expansion and add to the production of toxic polyglutamine protein [255].

### Potential impact on splicing factors

If repeat expansion sequences can mimic the binding motif of splicing regulators, they could recruit splicing factors and affect splice site selection. In DM1 the MBNL family of splicing factors and CUG binding proteins (CUGBPs) have an affinity for repetitive CUG and CAG sequence. Although the splicing of DM1 protein kinase (DMPK) mRNA does not appear to be affected by the CUG repeat expansion that it contains, the splicing pattern of an antisense transcript across the *DMPK* repeat, which contains a CAG repeat, appears to be altered by the expansion [105]. In HD the expanded CAG repeats have been proposed to interact with the splicing factor SRSF6, which is believed to contribute to altered splicing to generate truncated repeat-containing huntingtin mRNA [255].

Repeat expansion sequences in xtrRNA could also alter splicing by recruiting factors that are not typically involved in splicing. These factors might modulate splice site selection or spliceosome activity by changing local ribonucleoprotein (RNP) structure or access to splice signals [67]. The repetitive structural nature of repeat expansions could also sterically hinder access to splice signals, depending on their proximity to splice enhancer or silencer elements. Alternative splicing is a complicated interplay of modular protein and RNA interactions that are difficult to predict at present and local sequence and context will likely be important for understanding the impact of expanded repeats on splicing [14].

### Therapeutic approaches to control xtrRNA transcription and splicing

Characterizing the effect of microsatellite expansions on transcription and splicing will directly benefit therapeutic approaches for repeat expansion disorders. Proof-of-principle methods to locally disrupt the interactions of xtrRNA at repeat expansion loci, such as R-loops, have been demonstrated for FXS and FRDA using small molecules and nucleic acids [44, 177]. Disrupting the interaction of Spt4 and Spt5, or modulating Spt4 function, could provide a therapeutic avenue for a number of repeat expansion disorders by reducing xtrRNA expression. This has been demonstrated for CAG and GGGGCC repeat expansions [159, 186] and might be particularly valuable in disorders exhibiting bidirectional transcription across the repeat expansion. For splicing-based therapeutics, blocking inclusion of repeat expansion-containing introns, such as with splice-modulating antisense oligonucleotides or small RNAs, could prove to be useful for disorders like FRDA and C9FTD/ALS.

With the emergence of gene editing technologies, the direct removal of repeat expansions from the genome may also be possible. Removal of genomic repeat expansions could eliminate the possibility of xtrRNA expression or reverse repressive epigenetic states. Careful SNP selection followed by targeting with CRISPR-Cas9 has been shown to block promoter function and silence the mutant expanded allele in HD [215] or completely delete large portions of the mutant HD allele [265]. Targeting CRISPR-Cas9 to sequences flanking the CTG repeat in DM1 also caused large repeat deletions [299]. In model cells of DM1, CRISPR-Cas9 was used to introduce a poly-A signal upstream of the CTG expansion in the DMPK gene to prevent CUG repeat transcription, which led to a reversal of molecular disease [318]. While potential CRISPR-based therapeutics are exciting, precautions must be taken to address potential pitfalls and challenges like off-target effects, delivery, and cell-type specific mechanisms of DNA damage repair [16, 54, 71, 85, 229, 238, 240, 322].

## Structure, protein interactions, and localization of xtrRNA

### Structure of xtrRNAs and targeting with small molecules

During and after the synthesis and processing of xtrRNA, the repetitive RNA will fold into repetitive and unique structures and interact with proteins that have an affinity for its sequence or structure. Watson-Crick pairing apparently dominates folding since all atomic resolution investigations of disease-associated xtrRNA to date, including CAG, CUG, CCG, CGG, CCUG, AUUCU, and CCCCGG, are imperfect A-form-like duplexes [39, 62, 147, 234, 341]. These structures possess repeating units of Watson-Crick and mismatch paired nucleotides [147, 341]. While some studies have identified G-quadruplexes or tetraplexes [45, 79, 194, 247], other reports suggest that xtrRNA either do not form quadruplexes or are transient and interconvert readily with Watson-Crick paired conformations, especially as the number of repeating units increase [62, 108, 281, 329, 341]. Some reports of tetraplex structure may be the result of unusual interactions like dimerization between imperfect repeat duplex RNA, as was observed for CGG repeat RNAs [103]. Convincing evidence for the presence or biological significance of RNA G-quadruplexes inside human cells is still lacking [20, 107, 164, 194], therefore direct roles for quadruplex RNA in repeat expansion disease remain unclear.

Available structures of short repeat RNAs reveal A-form-like conformations with unique mismatches that may be targeted with artificial molecules to selectively bind repeat expansion RNA structure. Small molecule screening and structure-guided synthesis have experimentally identified a variety of small molecules that can bind xtrRNA, such as the CUG, CCUG, CGG, and GGGGCC repeat RNAs associated with DM1, DM2, FXS or FXTAS (Fragile X-associated tremor/ataxia syndrome), and C9FTD/ALS, respectively [38, 39, 226, 281, 292, 314, 325]. These molecules have been shown to stabilize repetitive structure or disrupt protein binding, which can correct molecular disease markers like nuclear RNA foci and repetitive polypeptide translation, or improve pathology in cells and animal models. Although promising, their eventual therapeutic application will need to demonstrate exquisite specificity for the RNA target, minimal non-specific interactions, and pharmacologic safety and efficacy [102, 252].

### Protein interactions and localization of xtrRNA

Both sequence specific and structure specific interactions likely underlie protein binding to xtrRNA. The repetitive nature of xtrRNA can result in multiple tandem binding sites for proteins. In DM1, the disease-associated xtrRNA contains hundreds or thousands of CUG repeats that bind and recruit possibly as many copies of MBNL-1 protein and potentially other CUG-binding proteins [173, 197, 235]. MBNL-1 recognizes CG dinucleotides separated by 1-17 nucleotides [92], which include motifs in pre-mRNA where MBNL-1 helps to regulate splicing [245]. Examples include the pre-mRNA of sarcoplasmic/endoplasmic reticulum Ca^2+^-ATPase 1 (SERCA1), which contains several YGCU(U/G)Y motifs downstream from exon 22. MBNL-1 usually interacts with these motifs to cause inclusion of exon 22 but in DM1 exon 22 is excluded during splicing [118, 34]. Blocking the interaction of proteins with DM1 xtrRNA by using morpholino oligonucleotides rescued splicing defects and molecular pathology [310]. Thus, a major contributor to disease mechanism in DM1 is the sequestration of splicing factors, particularly MBNL proteins.

A number of diseases are characterized by binding of specific proteins to xtrRNA or colocalization of proteins with xtrRNA focal aggregates (Table 1) [327]. These include proteins like MBNL-1 in DM1, DM2, HD, spinocerebellar ataxia 3 (SCA3), SCA8 and HDL2 [197, 282, 327], hnRNP K in SCA10 and C9FTD/ALS [46, 311], Pur-α, hnRNP F and SRSF2 in C9FTD/ALS [47, 108, 319], and Sam68 and hnRNP A2/B1 in FXTAS [262, 276]. As such, protein interactions with xtrRNA play key roles in disease mechanism and are expected to be important mediators of aberrant xtrRNA localization and aggregation [214]. Foci containing xtrRNA are believed to be the result of RNA-binding protein sequestration that can functionally deplete those proteins and partially protect the xtrRNA from degradation [214, 327].

Sequence specific interactions may not entirely explain xtrRNA localization or foci formation. While certain proteins that prefer to bind G-rich sequence, like hnRNP H/F, have been found to associate strongly with the GGGGCC repeats of C9FTD/ALS, other interacting proteins do not appear to have strong GGGGCC sequence-binding specificity, such as ALY/REF, SC-35, SF2, and nucleolin [47, 108, 175]. Imperfect A-form-like duplexes, or duplexes inter-converting with tetraplex conformations, may attract proteins that recognize the unique structures of xtrRNA rather than the specific sequence. Glycine-arginine-rich (GAR) proteins containing RGG/RG motifs, for example, are believed to recognize the structure of their nucleic acid partners rather than sequence [289]. The GAR domain-containing proteins FUS (fused in sarcoma), FMRP, and hnRNP U all recognize structured guanine-rich RNA sequences with an apparent preference for transitions between canonical duplexes and non-canonical structures like quadruplexes [233]. One explanation for foci is that proteins bind specifically to repeat sequence or structural elements of xtrRNA and then seed aggregation that recruits additional secondary interacting factors. Thus, xtrRNA may form foci by either merging with existing nuclear bodies or else establishing their own novel versions of RNA granules. While focal aggregation of xtrRNA can be detrimental to sequestered protein function, it may also protect the cell by preventing nuclear escape and translation of repeat RNAs [150].

### xtrRNA localization and membrane-free cellular organelles

Whether there is a specific localization pattern of xtrRNA inside cell nuclei is not entirely clear. Foci might be expected to nucleate at the site of transcription. DMPK mRNA usually localizes to SC-35 splicing speckles after transcription. However, when containing CUG repeat expansions, the DMPK mRNA has been shown to localize peri-transcriptionally outside of SC-35 splicing speckles [274]. RNA containing CAG, CUG and GGGGCC repeats were also shown to localize to SC-35 splicing speckles and nuclear speckles [132, 295]. However, in other studies the xtrRNAs, specifically CUG and CGG RNAs, appeared to form foci stochastically [243, 280]. Live cell imaging of Spinach2 aptamer-tagged CGG repeat xtrRNA revealed rapid aggregation and formation of very stable foci [280]. CGG xtrRNA foci were additionally found to be mobile and dynamic and colocalized with Sam68 protein. They migrated around the nucleus over time and could be seen to merge into larger foci or disaggregate into smaller foci. Live cell imaging of CUG repeat xtrRNA tagged with the MS2-GFP system found similar effects for aggregation, foci formation and dynamics [243]. CUG repeat RNA foci formation depended on the presence of MBNL-1 protein. In live-cell experimental approaches the xtrRNA is likely to be over-expressed from an artificial genetic context and may not represent the true dynamics or localization of endogenous repeat expansions. Nonetheless, live and fixed cell imaging have revealed that xtrRNA foci are dynamic, stable aggregates that likely depend on protein interactions and may co-localize with known nuclear bodies.

Nuclear bodies can be built around RNA and the molecular forces that govern nuclear body formation may help explain xtrRNA foci formation and localization. For example, nuclear paraspeckles depend on the long noncoding RNA NEAT1 (nuclear paraspeckle assembly transcript 1) [321]. Nuclear bodies are essentially membrane-free organelles that are held together by transient or dynamic protein-protein and protein-RNA interactions. These interactions collectively provide a type of phase separation to organize and compartmentalize cellular processes [336]. It was recently demonstrated that CAG, CUG and GGGGCC repeat containing RNAs form soluble aggregates with sol-gel phase separation properties and behave similar to liquid-like droplets [132]. These properties were dependent on the repeat expansion length and base-pairing interactions. In contrast, CCCCGG repeats did not form phase transitions, suggesting that not all xtrRNA will possess these properties. Interestingly, guanine-rich nucleic acids are less soluble than other nucleic acids and appear to be intrinsically aggregate-prone apart from protein, especially when packing into quartets or higher-order quadruplex structures [21, 89, 179]. The disruption of membrane-free organelles, which are abundant in the nucleus, is linked to disease [198, 228, 272]. In fact, the disruption of membrane-free organelle assembly and dynamics by repetitive poly-glycine-arginine (poly-GR) and poly-proline-arginine (poly-PR) translation products has emerged as a leading molecular disease mechanism for C9FTD/ALS [165, 174, 182]. Association of certain proteins with xtrRNA, dependent upon RNA sequence and structure, may strongly influence the subsequent localization of xtrRNA with membrane-free cellular compartments.

## Abundance and turnover of xtrRNA

### Abundance of foci-forming xtrRNA

Understanding the biology of an RNA includes knowing the effective concentration or abundance of that RNA and its turnover and decay pathways. Three current studies highlight the importance of characterizing cellular xtrRNA abundance. The cellular abundance of CUG repeat-containing transcripts was recently measured using transgenes and endogenous DMPK RNA in mouse models of DM1 and human tissues from DM1 patients [104]. Surprisingly, a large 1000-fold discrepancy for transcript number was discovered across mouse models. In human samples only a few dozen DMPK mRNA molecules were detected per cell, with only half of those expected to contain the repeat expansion. In a similar study looking at the abundance and processing of an antisense transcript across the *DMPK* repeat expansion, only a handful of repeat containing antisense transcripts were quantified per cell [105]. Quantification of the repeat-containing intron of C9ORF72 in C9FTD/ALS patient cells found only a few copies per cell, concluding that each foci might be composed of as few as one xtrRNA transcript [188]. Therefore, one or a few copies of xtrRNA may be enough to generate focal aggregates. Importantly, the stochastic nature of foci formation, where many cells contain no foci but some contain several, suggests that there may be a disproportionate contribution to disease for xtrRNA at the individual cell level [188]. These reports indicate that knowing the number and type of xtrRNA species inside of cells will be important for correct interpretation of data and for understanding the role of xtrRNA in disease.

### Nuclear xtrRNA retention and surveillance mechanisms

**Fig. 3:**
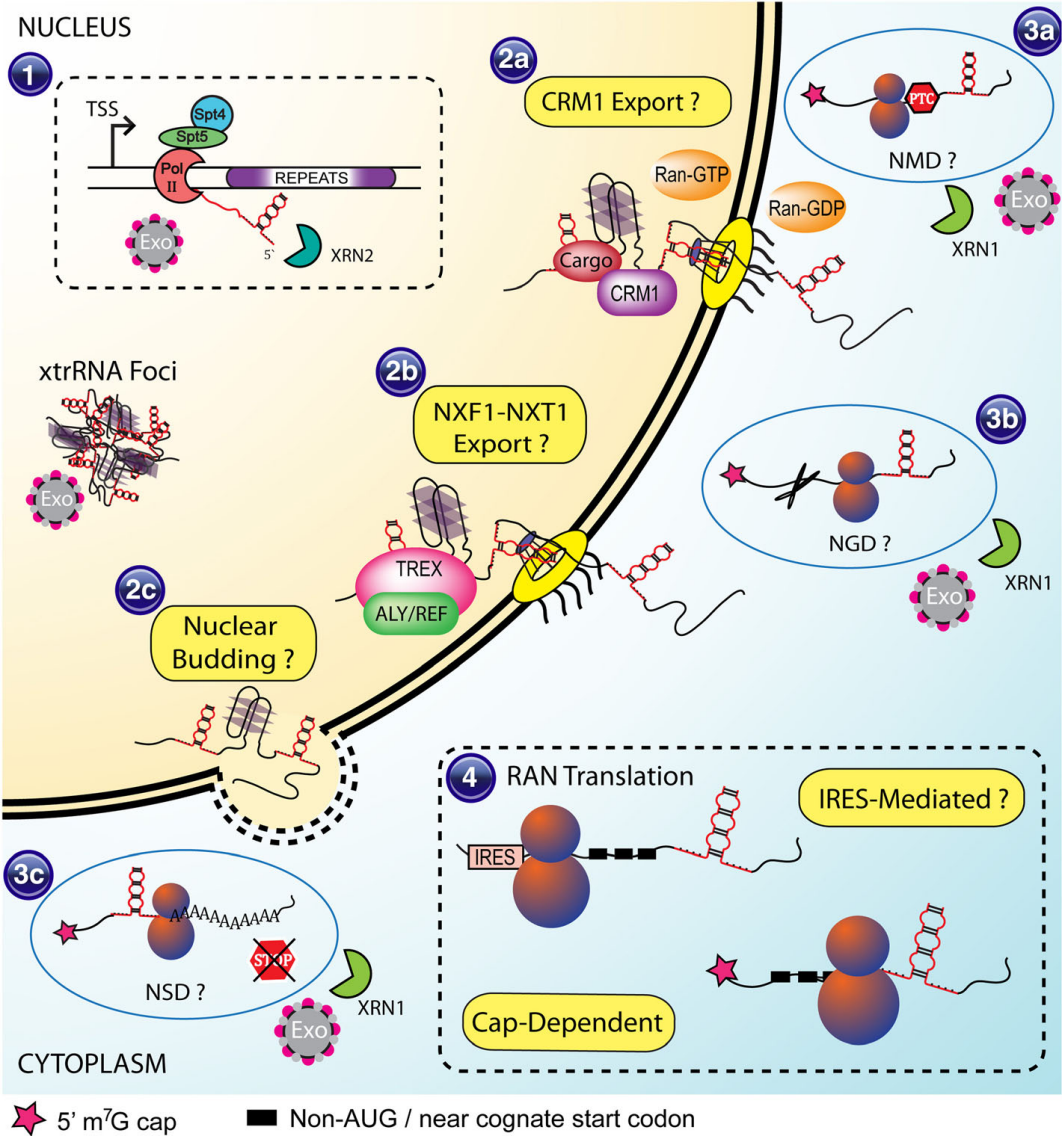
Possible mechanisms of nuclear and cytoplasmic RNA surveillance, nuclear export, and translation of xtrRNA. RNA containing large repeat expansion sequences may be subject to nuclear RNA surveillance mechanisms, including degradation by the nuclear exosome (1) or the XRN2 5'-3' exoribonuclease (1). Export of xtrRNA likely involves bulk mRNA transport via NXF1 (2b), but may also include alternative mechanisms like CRM1-mediated export (2a) or possibly nuclear envelope budding (2c). Cytoplasmic RNA surveillance mechanisms that may control xtrRNA levels and translation include nonsense-mediated decay (NMD) (3a), no-go decay (NGD) (3b), or nonstop decay (NSD) (3c). Translation of xtrRNA is likely to follow canonical cap-dependent translation (4), especially when repeat expansions are embedded in normal coding regions of an mRNA, but may potentially involve internal ribosome entry site (IRES)-like mechanisms (4). RAN translation has been shown to be cap-dependent for some repeat expansions, but complete mechanistic details remain to be determined

The nuclease enzymes primarily responsible for degrading nuclear RNA are the exosome complex (3'-5' exoribonuclease activity) and 5'-3' exoribonuclease 2 (XRN2) [67]. These enzymes act as part of a nuclear RNA quality control and surveillance pathway that monitors transcription, splicing, and 3'-end formation of pre-mRNAs, as well as their packaging into mRNP particles (Fig. 3) [67, 146, 338]. Instead of degradation, these pathways can also signal for retention of aberrant transcripts in the nucleus, typically at the site of transcription [52, 220] but sometimes near nuclear pores [67]. Retention at the site of transcription is coupled to nuclear exosome activity, particularly the Rrp6p subunit [66, 220]. The TPR protein, a mammalian ortholog of yeast Mlp1/2p, is implicated in retention at nuclear pores for mRNAs with retained introns that normally exit the nucleus through the nuclear export factor 1 (NXF1) pathway [49]. Both of these mechanisms may be relevant to xtrRNA, especially when repeat expansions are found in retained introns [43, 110, 227].

Surveillance mechanisms are also related to transcription or splicing of xtrRNA since these might be expected to trigger degradation [67, 146]. However, the existence of foci and nuclear export of xtrRNA argue that surveillance mechanisms are incomplete or inefficient for xtrRNA removal. At present it is unknown how many molecules of any repeat expansion-containing RNA are synthesized versus how many survive to form foci or exit the nucleus for translation. It is likely that repeat expansion-containing RNAs survive due to protection by protein binding, such as hnRNP proteins [125, 327]. Alternatively, factors responsible for recruiting RNA to the nuclear exosome, such as the TRAMP (Trf4/Air2/Mtr4p Polyadenylation) complex or NEXT (nuclear exosome targeting) complex, are unable to efficiently recognize and bind the xtrRNA [146, 259]. Thus, xtrRNA transcripts may escape degradation if they appear as "normal" mRNPs, having undergone proper RNA processing like capping, splicing and polyadenylation and are associated with appropriate post-processing factors.

### Turnover and decay of xtrRNA

An unanswered question remains as to whether foci might contain partially degraded fragments of repeat RNA in addition to larger intact transcripts. A case in point is C9FTD/ALS where the microsatellite expansion occurs in an intron but nuclear RNA foci and cytoplasmic translation are both observed. When introns are spliced out of pre-mRNA transcripts they are typically destined for rapid degradation unless they contain a stably folding RNA element or recruit RNA binding proteins [115]. Examples include small nucleolar RNAs and microRNAs [56, 203]. It is possible that the structures that repeat expansion RNAs form, as well as the proteins that they bind, allow them to persist, accumulate, and aggregate as foci [3]. At present the exact type and number of xtrRNA species that are trapped in foci versus free and soluble for any disease is unknown. It is also not known whether partially degraded xtrRNA fragments are stable enough to accumulate. Furthermore, there is no distinction between what are the major species or which are nuclear versus cytoplasmic.

When mRNAs that contain microsatellite repeat expansions reach the cytoplasm they may encounter additional quality control mechanisms designed to eliminate aberrant mRNA and prevent its translation. These include nonsense-mediated decay (NMD), no-go decay (NGD), and non-stop decay (NSD) (Fig. 3) [267]. NMD recognizes premature stop codons through possibly multiple mechanisms. NMD can be triggered if exon-junction complexes (EJCs), which are deposited near splicing junctions, are encountered downstream of a stop codon [172, 199]. Another mechanism of NMD may involve the relative length of sequence 3' to the stop codon [4, 172]. Repeat expansions could conceivably alter the positioning of a stop codon or extend the 3' UTR region and trigger NMD. When ribosome translation is significantly slowed or stalled then NGD can be triggered. Stalling is thought to be initiated by unusually stable RNA structures or protein-binding motifs and result in endonucleolytic cleavage [63]. Since repeat expansions are believed to fold into stable hairpins or tetraplexes they could possibly trigger NGD. In the case of NSD, the ribosome can become stuck on mRNA that does not possess a stop codon. These complexes must be resolved by cleavage and degradation of the mRNA [82, 302]. NSD could become activated if repeat expansions alter the reading frame or cause loss of the stop codon, such as via mis-splicing.

For xtrRNA embedded in mRNAs, either as exons or introns, mRNA surveillance mechanisms should act to reduce translation. Activation of these pathways will lead to degradation by the cytoplasmic version of the exosome and XRN1, a 5'-3' exoribonuclease. Accessory factors that guide cleavage include the Ski complex (composed of Ski2, Ski3, and Ski8 proteins) and the Ski7 protein [5, 267]. However, similar to nuclear RNA surveillance mechanisms, the cytoplasmic pathways seem unable to detect or completely remove xtrRNA and prevent translation. Methods to enhance RNA surveillance mechanisms might represent reasonable targets for therapeutic intervention. For example ataluren (PTC124) is a small molecule drug that increases NMD and might sensitize mRNA surveillance to repeat expansions [73].

Some studies have uncovered potential RNA turnover mechanisms associated with repeat expansions. In a recent RNAi screen several RNA processing factors were identified as suppressors of toxicity in *C. elegans* expressing a (CUG)_123_ repeat expansion in a 3'-UTR of GFP [88]. These factors included RNases, helicases and RNA binding proteins that, when knocked-down, caused increased toxicity and enhanced nuclear foci formation. A nuclear pore complex (NPC) protein, *npp-4*, was also a suppressor in this screen. Interestingly, *smg-2*, a conserved helicase and central component of the NMD pathway, was a strong suppressor. Knock-down of NMD resulted in a several-fold increase in GFP-(CUG)_123_ RNA expression levels and increased GFP translation. The substantial increase in 3'-UTR GC-content was identified as the likely trigger for NMD of the GFP-(CUG)_123_ RNA [88]. *Smg-2* was also identified as a suppressor of poly-glutamine aggregation previously, likely through NMD of aberrant repeat-containing HTT transcripts [328]. These results provide one example of the role of RNA turnover in controlling toxicity of xtrRNA in repeat expansion disorders.

## Nuclear export and translation of xtrRNA

### Canonical mRNA export pathways

RNA export from the nucleus involves several distinct pathways depending on the RNA and the various protein factors that constitute the RNP particle [273]. For nuclear mRNA there are two main export pathways: NXF1-mediated and chromosome region maintenance 1 (CRM1)-mediated, although NXF1 is the primary transport system for bulk mRNAs (Fig. 3) [60]. Both mechanisms rely on adapter proteins to specify the RNA cargo for export. Many of the factors required for successful export are deposited co-transcriptionally and post-transcriptionally during mRNP assembly [22]. The C-terminal domain of RNA Pol II serves as a docking platform for a wide variety of mRNA processing and mRNP assembly proteins and plays critical roles in establishing mRNP composition [139]. Correct processing of mRNA, such as capping, splicing and 3'-end formation, determines the ability of these factors to bind mRNA and assemble export-competent mRNP particles [36, 60, 335].

The mRNA-associated adapter proteins and complexes represent a complicated matrix of possible interactions that dictate export efficiency [22, 60]. NXF1 can specifically bind the constitutive transport element found in some mRNAs and viral RNAs, like that of the Mason Pfizer monkey virus, to directly facilitate export [101, 178]. However, for bulk cellular mRNA transport NXF1 uses adapters like TREX (transcription export complex) [60, 111]. Although the NXF1 protein interacts loosely with RNA, TREX helps mediate specific binding through its subunit ALY/REF [124], a protein previously reported to interact with *C9ORF72* GGGGCC RNA repeats [47]. TREX associates with mRNA during synthesis and processing via mRNA capping and splicing events [138, 204] and appears to be primarily recruited to the 5' ends of mRNAs in human cells via interaction between ALY/REF and the cap binding complex component CBP80 [36]. In addition to ALY/REF, TREX is composed of the THO complex, CIP29, and UAP56, a component of the EJC [37, 53, 157]. For repeat expansion disorders, NXF1 seems to be the most likely pathway since disease-associated xtrRNA are transcribed from coding gene loci and TREX is deposited onto mRNAs early during transcription [204].

### Nuclear export of xtrRNA

A recent study connected NXF1 transport of C9FTD/ALS intronic xtrRNA via interaction with the export adapter SR-rich splicing factor 1 (SRSF1) [110]. SRSF1 appeared to interact and colocalize with C9ORF72 xtrRNA. Depletion of SRSF1 prevented neurodegeneration in a fly model and suppressed cell death in patient-derived motor neurons and astrocytes. Depleting SRSF1 or preventing interaction with NXF1 inhibited nuclear export of repeat-containing *C9ORF72* transcripts and blocked RAN translation. Thus, SRSF1 might serve as a therapeutic target in C9FTD/ALS. This report highlights the value of understanding RNA biology in the context of repeat expansion disorders.

Most disease-associated xtrRNA is embedded in exonic or untranslated regions (Table 1) and therefore likely exits the nucleus via mRNA export pathways. CRM1 exports proteins and their associated RNAs via interaction with nuclear export signal sequences and Ran-GTP (Fig. 3) [60, 74, 77]. CRM1 interacts directly with the NPC at the nuclear periphery and commonly exports noncoding RNAs like spliceosomal RNA (snRNA) [10, 74, 131]. There is no reported RNA binding affinity of CRM1 so selective export of mRNAs depends on the RNA-binding properties of its cargo proteins [60]. Export of xtrRNA by CRM1 might only require that the repeat expansion sequence or structure somehow recruit a CRM1 cargo protein.

Export of intronic xtrRNA would be expected to require aberrant splicing that resulted in its retention in mRNA, as has been implicated for the intronic C9FTD/ALS repeat expansion [227]. Alternative export pathways exist but seem unlikely given their very specific nature. For example, transfer RNA (tRNA) undergoes multiple maturation phases that cumulatively result in two separate import and export steps [273]. These export pathways involve specific RNA-protein interactions, such as EXP-t and EXP5 [8, 29], that are unlikely to mediate xtrRNA export. For any export pathway through the NPC, xtrRNA must somehow establish RNP complexes that pass the requisite tests for licensing of export.

Nuclear exit of RNP granules, such as nuclear xtrRNA foci, might also be possible through nuclear envelope budding (Fig. 3). This mechanism involves TorsinA, nuclear lamina, and other uncharacterized factors. Nuclear budding was discovered as part of the nuclear egress mechanism of large nucleocapsid particles of Herpes viruses [55, 78, 200, 221, 277]. Nuclear envelope budding has been found to be a natural process for nuclear release of large RNP complexes during development of neuromuscular junctions in *Drosophila melanogaster* [135, 277]. However, knock-out of TorsinA in HeLa cells had little impact on Herpes virus production [294], suggesting alternative factors or mechanisms in human cells. If xtrRNA is exported by nuclear envelope budding it would have to mimic specific RNP granule formation that elicits nuclear envelope budding, which at present involves mechanisms that are largely uncharacterized [78].

### Translation of xtrRNA

If xtrRNA can successfully exit the nucleus it is a potential candidate for translation. However, mRNAs that contain expanded tandem repeats are possibly the only practical source for translation of repeat expansion polypeptides since they contain the prerequisite sequence elements and protein factors to mediate canonical cap-dependent translation. These typically include 5' cap structures, bound eukaryotic initiation factors (eIFs), a poly-A tail, and appropriate mRNP complexes like the EJC [116, 301, 315]. Translation of xtrRNA sequence embedded within the coding exon of a gene, such as is found in SBMA, HD, DRPLA (dentatorubral-pallidoluysian atrophy), OPMD (oculopharyngeal muscular dystrophy) and several of the SCA disorders (Table 1), are translated by canonical mechanisms. Most repeat expansions form stable secondary structures that have been shown to reduce the amount of overall translation by presumably inducing stalling, frame-shifting or abortive translation [72, 222, 244, 313]. In contrast, the specific binding of MID1 protein to huntingtin mRNA, which contains CAG repeat expansions, has been reported to enhance translation and lead to greater levels of aberrant protein [160]. This mechanism has also been proposed to enhance translation of other CAG repeat expansion-containing genes that cause disease [98]. Canonical translation of repeat expansions that are found in-frame in coding sequences is expected to generate otherwise normal protein that simply contain long tracts of repetitive polypeptide [296].

### Repeat-associated non-AUG translation

The translation of noncoding xtrRNA irrespective of a canonical start codon was recently discovered and termed repeat-associated non-AUG (RAN) translation [42, 96, 290, 339]. Repeat expansion diseases where this mechanism has been observed now include SCA2, SCA8, SCA31, HD, FXTAS/FXPOI, and C9FTD/ALS [9, 13, 27, 96, 129, 218, 261, 290, 339, 340]. RAN translation of xtrRNA sequence can occur in many contexts, including repeat expansions found in untranslated regions, retained introns, and even those embedded in coding exons [96]. The mechanisms of RAN translation remain poorly understood and could involve several scenarios, possibly even internal ribosome entry site (IRES)-like mechanisms (Fig. 3) [96, 339]. For the CGG repeats of FMR1 that cause FXTAS a more straightforward mechanism is emerging. In this case, RAN translation is m^7^G cap-dependent where a pre-initiation complex scans the RNA looking for a start codon [96, 141]. When the CGG repeats are present and stable structures are presumably encountered then stalling occurs and significantly enhances the ability of the ribosome to select a near-cognate start codon, or possibly any codon, to initiate translation [141, 154, 158, 339]. A similar mechanism is favored for the CAG and CUG repeats of sense and antisense transcripts in SCA8 [339]. This mechanism is proposed to allow translation initiation upstream of a repeat expansion in multiple reading frames [42, 96]. The sequence context, such as the leader sequence during scanning, the types of potential near-cognate start codons, and the repeat expansion sequence and size all appear to modulate the degree of RAN translation [12, 141, 261, 339].

The mechanism of RAN translation may be related to translation of upstream open reading frames (uORFs), a widespread phenomena revealed through high-throughput ribosomal footprint profiling [128]. RAN translation could even represent a specialized form of uORF translation that is triggered by stable xtrRNA structures. Both mechanisms can initiate at near-cognate start codons (although RAN translation may use other codons or other mechanisms, like frameshifting) and are influenced by surrounding sequence context that might impact RNA folding or protein interactions [96, 117].

Recent investigations have demonstrated that certain RAN translation products of C9FTD/ALS disrupt the function of membrane-free cellular organelles, such as stress granules, Cajal bodies and the nucleolus [174, 182]. These polypeptides seem to block the formation or critical interaction dynamics of membrane-free organelles and RNA granules, which are important for neuronal cell signaling and health [269, 288]. Transport of macromolecules through the nuclear pore complex depends on interactions that resemble membrane-free organelle structure [207, 219]. They are organized by dynamic protein interactions of low complexity domain proteins, including phenylalanine-glycine (FG) repeats, which may explain why certain C9FTD/ALS RAN translation products are reported to disrupt nucleocytoplasmic transport [80, 136, 264, 326]. RAN translation products can also aggregate and are implicated in the disruption of a variety of other pathways [12, 42, 96, 142, 286, 339].

Several important questions remain concerning the mechanisms of RAN translation. For example, how similar are the mechanisms of RAN translation across diverse repeat expansion and sequence contexts [42, 96]? RAN translation maybe a spectrum of related mechanisms based upon modulation of ribosomal scanning, translation initiation, and translation elongation [301]. RAN translation can initiate just upstream from the repeat expansion, but how often can RAN translation initiate within the repeat sequence itself [339]? *In vitro* and cell-based model systems suggest that RAN translation can proceed uninterrupted through an entire repeat expansion [141, 213, 339, 340]. Yet some expansions are massive in size. Therefore, how often do repeat expansions induce frame-shifting or possibly even early translation termination [313]? Also, what factors are unique to RAN translation? Finding answers to these mechanistic questions may be critical for developing future therapeutic molecules that can target and selectively block xtrRNA translation.

## Conclusion

RNA species that contain simple tandem repeat sequences occupy an underexplored world of RNA biology. Recent studies have begun to revisit the transcription and translation of repeat expansions. However, significant gaps remain for processes like cellular transport and turnover of xtrRNA. Placing repeat expansion disease mechanism studies in the context of current RNA biology will help reveal a better understanding of how the cell deals with xtrRNA and identify mechanisms unique to repeat expansions.

Investigations into the biology of xtrRNA promise to unlock new approaches to therapeutics. Transcription across repeat expansions has opportunities for therapeutic development, such as modulating the function of Supt4h. Likewise, translation of repeat expansions, especially RAN translation, may become more targetable as molecular mechanisms become better characterized and specific factors identified. Selectively blocking both the synthesis of xtrRNA or its translation are attractive therapeutic approaches since they could extrapolate to multiple repeat expansion disorders. Turnover of xtrRNA should become increasingly important since several potential therapeutic strategies employ targeted and selective degradation of repeat expansion-containing RNA, such as antisense oligonucleotides and small interfering RNAs [64, 134, 169, 239, 275].
